# Acute Pancreatitis Secondary to Newly Diagnosed Intraductal Papillary Mucinous Neoplasm

**DOI:** 10.7759/cureus.24526

**Published:** 2022-04-27

**Authors:** Brandon Wiggins, Fady Banno, Justin Miller

**Affiliations:** 1 Internal Medicine, Ascension Genesys Hospital, Grand Blanc, USA; 2 Gastroenterology, Ascension Genesys Hospital, Grand Blanc, USA

**Keywords:** case report, mrcp, gastroenterology, intraductal papillary mucinous neoplasm, acute pancreatitis

## Abstract

Acute pancreatitis (AP) is a common gastrointestinal cause of hospital admissions and is prevalent in the United States. AP etiologies include alcohol use, cholelithiasis, hypertriglyceridemia, hypercalcemia, autoimmune phenomena, medications, or idiopathic. Rarely, intraductal papillary mucinous neoplasms can cause AP, as we present in this case report.

## Introduction

Acute pancreatitis (AP) accounts for over 220,000 hospital admissions in the United States each year [1]. Acute and recurrent pancreatitis is caused by gallstones and alcohol use in 70-80% of patients in the western world [2]. Tumors, both benign and malignant, hypertriglyceridemia, hypercalcemia, trauma, infections, and hereditary or congenital abnormalities are among the other causes of acute or recurrent pancreatitis. It is interesting to note that tumors, both benign and malignant, are documented to cause recurrent pancreatitis in about 9% of patients [3]. Pancreatitis has also been commonly reported with pancreatic ductal adenocarcinoma [4,5].

Intraductal papillary mucinous neoplasms (IPMNs) were first described in the 1980s, and since then, the diagnosis is increasingly reported worldwide, currently accounting for 5% of pancreatic neoplasms [6-11]. Due to advancements in radiologic imaging, early detection has become more frequent. IPMNs arise from the pancreatic duct and may be found around the pancreas. They are usually described in three forms, namely, main pancreatic duct (MD-IPMN), side branch (SB-IPMN), or combined. Though the rate of AP secondary to IPMN is not directly defined in the current literature, it is one of the rarest etiologies of the disease process [12].

AP associated with IPMN is often an indication for surgery according to published guidelines, as reoccurrence is likely, increasing morbidity and mortality [12-14]. Cyst size is used for the surveillance of IPMN. If the cyst size is less than 1 cm, magnetic resonance cholangiopancreatography (MRCP) should be performed every two years for four years. If the cyst size is 1-2 cm, MRCP should be performed every year for three years, followed by every two years for four years. If the cyst size is 2-3 cm, MRCP/endoscopic ultrasound (EUS) is recommended every 6-12 months for three years, followed by every year for four years, with the interval increased once the size is stabilized. If the cyst is larger than 3 cm, MRCP alternates with EUS every six months for three years, followed by MRCP alternates with EUS every year for four years. The gap is increased once the cyst is stable in size. Surgery is recommended in patients with jaundice or AP secondary to IPMN, or if there is a significantly elevated serum carbohydrate antigen 19-9 (CA 19-9) level, presence of solid components within the cyst, over three IPMNs, main pancreatic duct dilation of > 5 mm, pancreatic duct dilation for MD-IPMN or by an obstructing lesion, cytology of pancreatic cancer, and the presence of high-grade dysplastic IPMN. These patients should be further evaluated by a multidisciplinary team [15].

The pathogenesis of AP due to IPMN is not well described in the literature. Some evidence suggests AP from IPMNs is mainly due to main duct obstruction by thick mucin. This leads to increase ductal pressure with the premature release of pancreatic enzymes, similar to other causes of obstructive pancreatitis [16].

## Case presentation

A 73-year-old female with a medical history of cirrhosis secondary to hepatitis B, hypertension, type 2 diabetes mellitus, chronic obstructive pulmonary disease (COPD), peripheral vascular disease, and anemia of chronic disease presented to the emergency department with epigastric pain. She described the pain as severe, radiating to the back, sharp in nature, constant in timing, and epigastric in location. She stated the pain was provoked by eating and was associated with decreased oral intake, nausea, non-bilious, non-bloody emesis, and night sweats for one week. She denied any history of drug, alcohol, or tobacco use. She also denied starting any new medications during the same period of symptoms. She stated that she was diagnosed with hepatitis B 20 years previously and was subsequently started on entecavir.

Laboratory results on presentation were significant for total bilirubin of 3.4 mg/dL, aspartate aminotransferase of 83 U/L with normal alanine aminotransferase, and lipase of 217 U/L. Of note, lipase level was 12 U/L three weeks prior on a separate admission. Triglycerides and calcium were within normal limits, and blood alcohol level was 0 mg/dL (Table 1).

**Table 1:** Initial laboratory results.

Laboratory values	Measured	Normal range
White blood cell count	9 K/cm^2^	4.5–11 K/cm^2^
Hemoglobin	11.4 g/dL	11–16.2 g/dL
Hematocrit	36.7%	36–46%
Platelet count	103 K/cm^2^	140–440 K/cm^2^
Serum sodium level	135 mmol/L	136–144 mmol/L
Serum potassium level	3.3 mmol/L	3.6–5.1 mmol/L
Serum chloride level	99 mmol/L	101–111 mmol/L
Total serum carbon dioxide	24 mmol/L	20–30 mmol/L
Serum blood urea nitrogen	7 mg/dL	8–26 mg/dL
Serum creatinine level	0.72 mg/dL	0.44–1.00 mg/dL
Anion gap	12 mmol/L	8–16 mmol/L
Serum glucose	245 mg/dL	70–99 mg/dL
Serum calcium level	9.4 mg/dL	8.4–10.2 mg/dL
Serum magnesium level	1.1 mg/dL	1.6–2.6 mg/dL
Serum phosphorous level	2.4 mg/dL	2.3–4.7 mg/dL
Total bilirubin	3.4 mg/dL	0.3–1 mg/dL
Serum albumin level	3.2 g/dL	3.5–5 g/dL
Aspartate aminotransferase	83 U/L	15–41 U/L
Alanine aminotransferase	26 U/L	14–54 U/L
Serum alkaline phosphatase	208 U/L	41–150 U/L
Triglycerides	98 mg/dL	<150 mg/dL
Serum lipase	217 U/L	22–51 U/L
Blood alcohol level	0 mg/dL	<10 mg/dL

**Figure 1:**
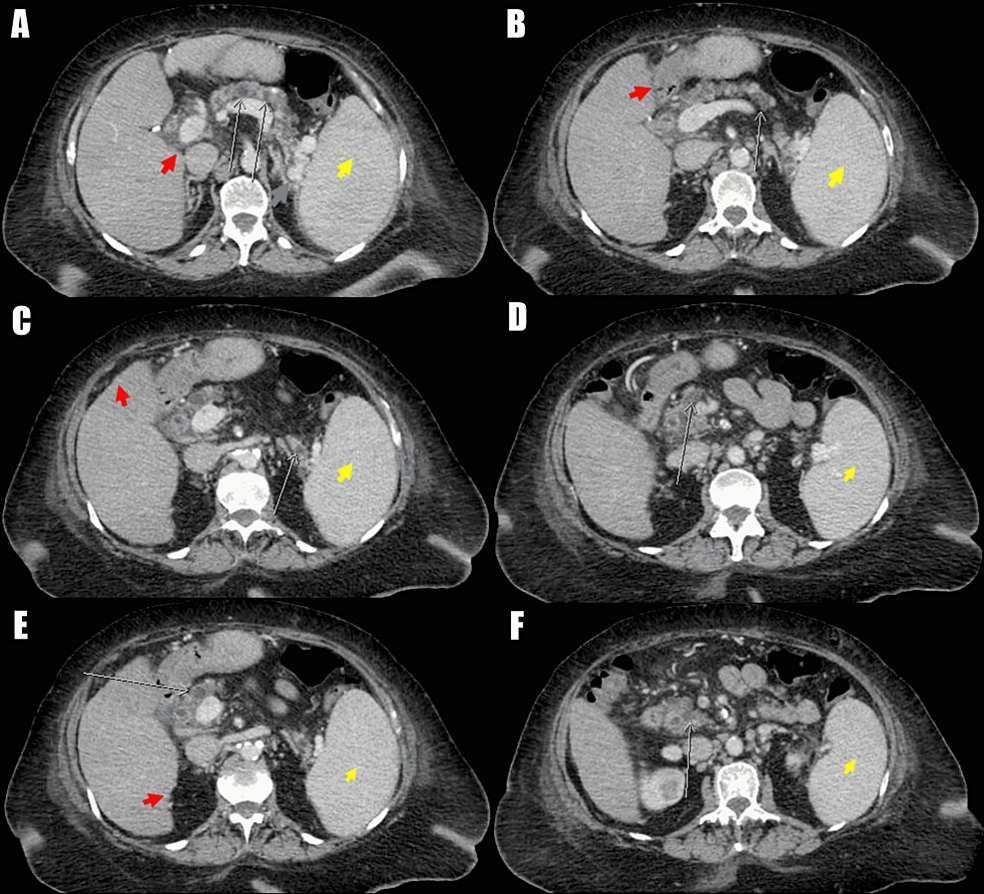
Computed tomography of the abdomen: transverse views. A: White arrows demonstrating intraductal papillary mucinous neoplasms within the pancreas. Red arrow demonstrating cirrhotic liver with collateral vessels and nodular border. Blue arrow demonstrating portal hypertensive collateral vessels. Yellow arrow demonstrating splenomegaly. B: White arrow demonstrating intraductal papillary mucinous neoplasms within the pancreas. Red arrow demonstrating cirrhotic liver with collateral vessels and nodular border. Yellow arrow demonstrating splenomegaly. C: White arrow demonstrating acute pancreatitis of the pancreatic tail. Red arrow demonstrating cirrhotic liver with collateral vessels and nodular border. Yellow arrow demonstrating splenomegaly. D: White arrow demonstrating intraductal papillary mucinous neoplasms within the pancreas. Yellow arrow demonstrating splenomegaly. E: White arrow demonstrating intraductal papillary mucinous neoplasms within the pancreas. Red arrow demonstrating cirrhotic liver with collateral vessels and nodular border. Yellow arrow demonstrating splenomegaly. F: White arrow demonstrating intraductal papillary mucinous neoplasms within the pancreas. Yellow arrow demonstrating splenomegaly.

**Figure 2:**
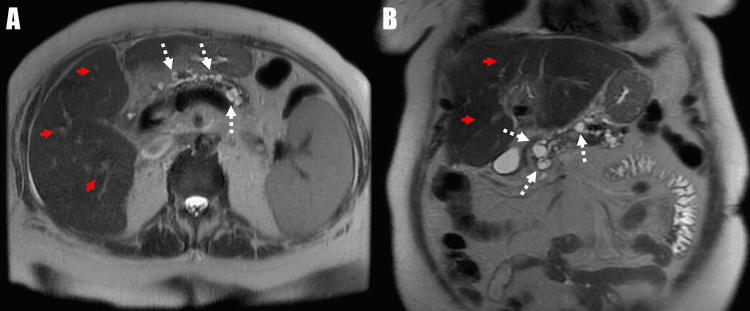
Magnetic resonance cholangiopancreatography: transverse and coronal view. A: White arrows demonstrating intraductal papillary mucinous neoplasms within the pancreas. Red arrows demonstrating cirrhotic liver with collateral vessels and nodular border. B: White arrows demonstrating intraductal papillary mucinous neoplasms within the pancreas. Red arrows demonstrating cirrhotic liver with collateral vessels and nodular border.

Computed tomography (CT) scan of the abdomen and pelvis with intravenous contrast demonstrated cirrhotic-appearing liver, significant variceal formation within the liver and esophagus, innumerable side branch pancreatic IPMNs, and inflammation of the pancreatic tail consistent with AP (Figure 1). Ultrasound of the abdomen showed evidence of cholecystectomy, cirrhotic changes, and variceal dilation of the left portal vein. Subsequently, MRCP was ordered and revealed evidence of portal hypertension, splenomegaly, perihepatic fluid, abdominal varices, and innumerable IPMNs, with the largest measuring 1.1 cm (Figure 2).

The patient was started on lactated ringers intravenous fluid, made nothing per os, and started on an opioid pain medication regimen. Within the next week, the patient’s pain resolved, and her diet was advanced. She improved and was discharged home with close follow-up at a tertiary care center for newly diagnosed IPMNs for surveillance and consideration for surgery.

## Discussion

It is important to recognize the epidemiological factors in patients with AP secondary to IPMN. Age, sex, alcohol consumption, and smoking history are among the most significant factors. IPMN presents more frequently in older patients (63.1 ± 11.0 years) and females with low-to-moderate alcohol consumption and smoking usage [17].

Currently, there are four different subtypes of IPMN, namely, gastric, intestinal, oncocytic, and pancreatobiliary. The pancreatobiliary subtype is more likely to progress to adenocarcinoma in 50% of patients. IPMN should be considered on the differential if patients present with recurrent AP or AP after exclusion of other common etiologies. Evaluation of other causes of pancreatitis is important, including cholelithiasis, medications, and sphincter of Oddi dysfunction. If the initial evaluation is negative, MRCP should be performed to evaluate for dilation of the duct of Wirsung [18].

The diagnosis of IPMN has substantially increased due to the development of more accurate imaging techniques [17]. IPMN is thought to be due to duct dilation and obstruction caused by mucin accumulation which ultimately forms multiple pancreatic cysts. Previously, IPMN was thought to be benign; however, some patients will develop abdominal pain resembling AP and others will develop pancreatic insufficiency resembling chronic pancreatitis [19]. Moreover, some IPMNs will eventually develop into adenocarcinoma, depending on size, consistency, and presenting symptoms. Due to prolonged occlusion of the main duct by large amounts of mucin, many patients develop pancreatic insufficiency and diabetes with weight loss [20].

It is possible to perform EUS with fine-needle aspiration to provide tissue for analysis. However, surgery is the mainstay option for treatment with excellent five-year survival [7].

Our patient presented with classic AP symptomatology with acute epigastric pain radiating to the back that was constant and stabbing in nature with provocation upon oral intake. The patient denied alcohol use. Both CT abdomen and MRCP excluded gallstone pancreatitis, and none of her medications were reported to cause pancreatitis in the medical literature. The patient did not have elevated triglycerides or calcium. MRCP proved invaluable in identifying the etiology of AP as countless IPMNs were discovered. Furthermore, IPMNs were absent on abdominal imaging one month prior.

## Conclusions

AP is a common gastrointestinal disorder that is treated by physicians frequently in outpatient and inpatient settings. AP has many different etiologies with different diagnostic modalities and treatments. When common causes are ruled out, more rare etiologies, such as IPMN, need to be considered. In the above case, we highlight the value in acquiring CT and MRCP to delineate a rare cause of AP.

Current medical literature is lacking in specific epidemiology factors such as prevalence, incidence, morbidity, and mortality with regards to AP secondary to IPMN. With obscurity surrounding this gastrointestinal disease in the literature, it is imperative for future studies to identify more accurate epidemiology factors to better understand this rare disease process.
